# Associations between Mixture of Perfluoroalkyl Substances and Lipid Profile in a Highly Exposed Adult Community in the Veneto Region

**DOI:** 10.3390/ijerph191912421

**Published:** 2022-09-29

**Authors:** Erich Batzella, Maryam Zare Jeddi, Gisella Pitter, Francesca Russo, Tony Fletcher, Cristina Canova

**Affiliations:** 1 Unit of Biostatistics, Epidemiology and Public Health, Department of Cardio-Thoraco-Vascular Sciences and Public Health, 35131 Padova, Italy; 2 RIVM-National Institute for Public Health and the Environment, 3720 Bilthoven, The Netherlands; 3 Screening and Health Impact Assessment Unit, Azienda Zero-Veneto Region, 35131 Padua, Italy; 4 Directorate of Prevention, Food Safety, Veterinary Public Health-Veneto Region, 30123 Venice, Italy; 5 Department of Public Health, Environments and Society, London School of Hygiene and Tropical Medicine, London WC1E 7HT, UK

**Keywords:** perfluoroalkyl substances, lipid, cholesterol, contaminated water, combined exposure, mixture, WQS, G-computation, BKMR

## Abstract

Background: Residents of a large area in the Veneto Region (Northeastern Italy) were exposed to drinking water contaminated by perfluoroalkyl substances (PFAS) for decades. While exposure to PFAS has been consistently associated with elevated serum lipids, combined exposures to multiple PFASs have been poorly investigated. Utilising different statistical approaches, we examine the association between chemical mixtures and lipid parameters. Methods: Cross-sectional data from the regional health surveillance program (34,633 individuals aged 20–64 years) were used to examine the combined effects of PFAS mixture (Perfluorooctanoic acid (PFOA), perfluorooctane sulfonate (PFOS), perfluorononanoic acid (PFNA) and perfluorohexane sulfonate (PFHxS)) on total cholesterol (TC), high-density lipoprotein cholesterol (HDL-C) and low-density lipoprotein cholesterol (LDL-C). Weighted Quantile Sum (WQS) regression, Quantile-based G-computation (Q-Gcomp) and Bayesian Kernel Machine Regression (BKMR) were used based on their ability to handle highly correlated chemicals. Results: We observed that each quartile increase in the WQS index was associated with an increase in the levels of TC (β: 4.09, 95% CI: 3.47–4.71), HDL-C (β: 1.13, 95% CI: 0.92–1.33) and LDL-C (β: 3.14, 95% CI: 2.65–3.63). Q-Gcomp estimated that a quartile increase in the PFAS mixture was associated with increased TC (ψ: 4.04, 95% CI 3.5–4.58), HDL-C (ψ: 1.07, 95% CI 20.87–1.27) and LDL-C (ψ: 2.71, 95% CI 2.23–3.19). In the BKMR analysis, the effect of PFAS mixture on serum lipids increased significantly when their concentrations were at their 75th percentiles or above, compared to those at their 50th percentile. All methods revealed a major contribution of PFOS and PFNA, although the main exposure was due to PFOA. We found suggestive evidence that associations varied when stratified by gender. Conclusions: The PFAS mixture was positively associated with lipid parameters, regardless of the applied method. Very similar results obtained from the three methods may be attributed to the linear positive association with the outcomes and no interaction between each PFAS.

## 1. Introduction

Per- and polyfluoroalkyl substances (PFAS), a large class of organic chemicals, have been extensively used in industrial and commercial products throughout the 20th and 21st centuries due to their unique physical and chemical characteristics [1]. Most PFASs are highly persistent in the environment, can bioaccumulate in human bodies and, as a result of widespread use, many PFASs are now defined as persistent environmental pollutants [2,3]. While exposure appears to have declined in the general population, nearly every human has detectable concentrations of some of these chemicals [4]. Legacy long-chain PFASs have long elimination half-lives in human serum on the order of years [e.g., >2.1 years for perfluorooctanoic acid (PFOA), >3.3 years for perfluorooctane sulfonate (PFOS), >4.7 years for perfluorohexane sulfonic acid (PFHxS) and >2.5 years for perfluorononanoic acid (PFNA)] [5].

Given the extensive bioaccumulation and persistence of PFAS, numerous epidemiological studies have found PFAS exposure to be associated with various adverse health outcomes, including increased cholesterol levels, immune system dysregulation and toxicity, liver and kidney disease, thyroid hormones disruption, developmental effects in children and reproductive effects [6]. A considerable number of epidemiological studies, including our previous work [7], conducted both on highly exposed populations as well as populations with background exposure, showed that exposure to individual PFASs, mainly PFOA and PFOS, is associated with altered lipid profiles [6]. However, only a few studies have examined the association between PFAS mixtures and lipid outcomes [8,9,10,11], taking into account the totality of chemical exposures simultaneously, which upon acting together may have additive, synergistic, antagonistic or potentiating effects [12]. All the above-mentioned studies referred to small communities (hundreds of subjects) with background levels of exposure.

Hence, the main objective of the present study was to address this research gap, examining the associations between exposure to a mixture of four PFASs, namely, PFOA, PFOS, PFNA and PFHxS, and serum lipid profile among a large sample of more than 34,000 highly exposed community residents in Italy. Residents of a large area in the Veneto Region (Northeastern Italy) were exposed to high concentrations of PFAS, particularly PFOA, via contaminated drinking water stemming from activities of a manufacturing plant since the late 1960s [13]. In response to the public concern, a health surveillance program was established to evaluate the relationship between PFAS and different health outcomes [7,14,15,16].

Given that no statistical mixture method is considered the gold standard [17], both Weighted Quantile Sum (WQS) regression, Quantile-based G-computation (Q-Gcomp) and Bayesian kernel machine regression (BKMR) were used and compared based on their ability to handle highly correlated measurements of PFAS to identify the impact of a mixture of chemicals on lipid parameters.

## 2. Materials and Methods

### 2.1. Participants and Study Design

A health surveillance program was launched in the Veneto Region, Italy, in January 2017 as a free, population-based screening program offered by the regional Health Service to the residents of 30 municipalities who were exposed to PFAS via contaminated drinking water for several decades until 2013, when waterworks were supplied with granular activated carbon filters leading to an abrupt reduction of PFAS concentrations (for more information please see [13]). The eligible group, which consisted of more than 100,000 residents born between 1951 and 2014, was invited to participate in the health survey through the Regional Health Registry and invitation letters were mailed to them. As part of the study, participants completed an interview questionnaire assessing socio-demographic characteristics, personal health history, lifestyle habits, and self-reported height and weight. Non-fasting blood samples were obtained for the convenience of the participants throughout the course of the day when they visited the clinic.

The current study’s population is a subset of the surveillance program target population aged 20–64 years at recruitment (*n* = 36,517 subjects, participation rate 61%). Owing to the fact that blood lipid levels may change during pregnancy, and because pregnancy can modify PFAS exposure, distribution or excretion, pregnant women (*n* = 394) were excluded from the analysis. Additionally, individuals who admitted to using statins and other cholesterol lowering medications, fibrates or red rice (a naturally occurring statin) were also excluded (*n* = 1325). Finally, 165 participants (less than 0.5%) who had missing data on the selected covariates were excluded, leaving a total of 34,633 subjects for analyses (Figure S1).

### 2.2. PFAS Quantification

Serum concentrations of twelve PFASs were quantified by using high-performance liquid chromatography–tandem mass spectrometry (HPLC-MS/MS) [Prominence UFLC XR 20 (Shimadzu) coupled to API 4000TM LC-MS/MS System (Sciex)]: perfluorooctanesulfonate (PFOS), perfluorooctanoic acid (PFOA), perfluorohexanesulfonic acid (PFHxS), perfluorononanoic acid (PFNA), perfluoroheptanoic acid (PFHpA), perfluorobutanesulfonic acid (PFBS), perfluorohexanoic acid (PFHxA), perfluorobutanoic acid (PFBA), perfluoropentanoic acid (PFPeA), perfluorodecanoic acid (PFDeA), perfluoroundecanoic acid (PFUnA) and perfluorododecanoic acid (PFDoA). Additional information regarding PFAS measurement is provided elsewhere [13].

The analytical method has been validated according to the UNI CEI EN ISO/IEC 17,025 regulation, and laboratory analyses included low- and high-concentration pooled quality control materials, standards, reagent blanks and study samples. The measuring range of the method was 0.5–500 ng/mL. The Repeatability of the method given as RSD of determined concentrations was below 10% for all measured PFAS at different levels and the accuracy of the method was between 80 and 120%. Limit of detection (LOD) for all PFAS was as low as 0.1 ng/mL and limit of quantification (LOQ) was 0.5 ng/mL [13]. Concentrations below the LOQ were imputed by dividing the LOQ by the square root of 2 prior to statistical analysis. PFASs detected in greater than 50% of participants were included in the main analyses, namely, PFOA (99.86%), PFOS (99.74%), PFHxS (96.89%) and PFNA (52.93%). The excluded PFASs were all detected in less than 20% of participants (Table S1).

### 2.3. Outcome Assessment

The assessments of lipid markers, including total cholesterol (TC), high-density lipoprotein cholesterol (HDL-C), and low-density lipoprotein cholesterol (LDL-C), were performed in three laboratories within the hotspots (contaminated municipalities as Arzignano, San Bonifacio and Legnago). The level of total cholesterol and HDL-C were measured by a direct enzymatic colorimetric test and units were recorded in mg/dL. When the levels of triglycerides were less than 400 mg/dL, the Friedewald equation was used to calculate the level of LDL-C. An external quality assurance program is consistently followed by the three laboratories.

### 2.4. Covariates

A directed acyclic graph (DAG) was used to identify sociodemographic, lifestyle and medical history data that could potentially bias observational associations of PFAS exposures with lipid profile [7], including age, body mass index (BMI) and time-lag between the enrolment and the beginning of the study as continuous variables, and smoking (non-smokers, current or former smoker), alcohol consumption (0, 1–2, 3–6, 7+ alcohol units per week), country of birth, education level, laboratory in charge of the analyses of serum lipids, and dietary habits (tertiles or quartiles of meat, fish/seafood, milk/yogurt, cheese, eggs, bread/pasta/cereals, sweets/snacks/sweet beverages and fruits/vegetables consumption) as categorical covariates. Additional details regarding the covariate and confounders selection have been described elsewhere [7].

### 2.5. Statistical Analysis

The serum concentrations of PFAS are expressed as geometric mean (GM), arithmetic mean (standard deviation, SD) and percentiles in total and stratified by gender (male, female). Serum PFAS levels were natural log-transformed to achieve a normal distribution and to improve the model fit. Spearman’s rank correlation (ρ) was calculated to evaluate pair-wise correlations between the PFASs.

Building upon our previous research [7], we ran single-chemical linear regression models using generalized additive models (GAMs), to examine independent associations between each PFAS and each lipid parameter and corroborate results on a population with a wider age range than the one previously analysed (20–64 years old instead of 20–39 years old). We also assessed the effect of the sum of all 12 measured PFASs on cholesterol levels (ΣPFAS), including those with detection rates below 50%. We modelled the chemical exposures as natural log-transformed (ln-PFAS) continuous variables. An interaction term between sex and ln-PFAS linear coefficient was also added to the models. We further categorized serum PFAS concentrations into quartiles. The established set of covariates was fully accounted for in all of the models (age, BMI, time-lag between the enrolment and the beginning of the study, sex, smoking habits, country of birth, alcohol consumption, education level, laboratory in charge of the analyses of serum lipids and reported food consumption).

#### PFAS Mixtures

We estimated effects of the mixture of four PFASs on lipids profile outcomes using three approaches: WQS regression, Q-Gcomp regression and BKMR. The main characteristics of the selected methods are listed in Table S2.

WQS regression summarizes the overall exposure to the mixture by creating a weighted linear index of correlated predictors that are weighted according to their strength of association with the outcome of interest. A detailed description of WQS regression can be found in Carrico et al. [18].

Prior to analysis, we split the dataset randomly into two subsets, a training set (40%) for estimating the weights in an ensemble step averaging results across bootstrap samples (100 bootstrap samples) and a validation set (the remaining 60%) estimating the coefficient mapped to the mixture, conditionally on the estimated weights.

We scored PFAS in quartiles and used a threshold of 0.25 (1/*p*, where p represents the number of exposure variables) for weights, as a guide to determine which PFASs have the most contribution to the overall mixture effect (chemicals of concern), as well as to discuss qualitatively their contributions in relation to each other. WQS regression assumes linear exposure–outcome associations across quantiles of each exposure and focuses inference on a single direction at a time with constrained optimization of the beta parameter. Effects can be estimated for both directions individually by conducting separate analyses, but in our case we constrained the analyses to positive direction.

We next applied a quantile G-computation (Q-Gcomp) approach, a recent extension to the WQS regression introduced as a generalization of the classical G-computation approach, where the regression is performed using quantized exposures. Further details on methodology can be found in Keil et al. [19]. Similar to WQS, the G-computation parameter quantifies the expected change in the outcome, given a one quantile increase in all exposures simultaneously, adjusted for the available confounders. The exposure to four PFASs is considered as an index that represents their joint value, after transforming them in “scores” taking on discrete values (quartiles, mirrorwise to WQS). This method shares the directional homogeneity assumption of WQS regression without any constraints of positive weights, redefining them to be both negative or positive; weights can be interpreted as the partial effect due to a specific exposure. G-computation can also overcome the linearity assumption of WQS by including polynomial terms for exposures, which makes it a valuable tool for identifying mixture with harmful constituents on a health outcome of interest [19], bringing certain advantages over traditional WQS. We defined as chemicals of concern PFAS contributing to the overall mixture effect with a positive weight that exceeded the threshold of 1/*p* (where p, in contrast with WQS, varied depending on the number of PFASs with estimated positive weight in each stratum).

We also assessed PFAS mixture association using BKMR analysis, which is able to elucidate the relation between PFAS and lipid profile allowing exposures to relate non-linearly and non-additively with the outcomes [20,21,22]. The core of the BKMR model is kernel machine regression (KMR), also called Gaussian process regression, which combines Bayesian and statistical learning methods to iteratively regress the exposure–response function using a Gaussian kernel function [23]. Overall mixture effects were displayed taking the mean value of the outcome when all four PFAS concentrations were at the 75th percentile compared to the mean value of the outcome when concentrations were at the 50th percentile, to makes results more readily comparable with the previous methods [20]. Further details on post-estimation visualizations and statistics from BKMR analyses can be found in Bobb et al. [20]. BKMR can also be used as a variable selection tool, identifying exposure variables that are important to the overall effect of the exposure mixture by estimating posterior inclusion probabilities (PIPs). Like WQS and Q-Gcomp weights, PIPs can be used to identify the importance of individual exposure variables to the overall mixture effect. However, unlike the other mixture model techniques weights, PIPs are not constrained to sum to 1.

Further, we assessed heterogeneity in effects by gender using sex-specific models for all lipid outcomes.

All analyses were fully adjusted for the established set of covariates described in the previous section.

Analyses were performed using the statistical and R [24]. We employed “gWQS” [25], “qgcomp” [19] and “bkmr” [20] packages to conduct WQS, Quantile G-computation and BKMR with R, respectively. Results with *p*-values < 0.05 were considered statistically significant.

## 3. Results

The study population comprised 18,320 (52.9%) females and 16,313 (47.1%) males, with a mean age of 39.95 years (SD 10.98, range 20–64). General characteristics of participants are reported in Table S3. Table 1 provides descriptive statistics on serum concentration of PFAS and lipid parameters. 

Serum levels of PFOA, PFOS, PFHxS and PFNA were on average and maximum values higher in males than females (*p*-value < 0.05). Among the four PFASs, PFOA levels were much higher (GM 32.76 ng/mL), followed by PFOS, PFHxS and PFNA (GMs 4, 3.86 and 0.5 ng/mL, respectively). As for lipid profile, the median serum concentration for TC, HDL-C and LDL-C were 187, 58 and 107 mg/dL, respectively. Levels of LDL were generally higher among males than females, and HDL-C was higher in females (*p*-value < 0.05).

There were significant correlations among the four PFASs (Table S4). The correlation between PFOA and PFHxS was the strongest (ρ = 0.90), whereas that between PFOS and the other two PFASs was less significant (ρ = 0.68 with PFNA, ρ = 0.65 with PFHxS and ρ = 0.60 with PFOA).

In the single PFAS models, each ln-increase in PFAS was associated with an increase of 1.83 mg/dL (CI 1.51–2.15) in TC for PFOA, 5.14 mg/dL (CI 4.56–5.72) for PFOS, 1.74 mg/dL (CI 1.36–2.13) for PFHxS and 6.61 mg/dL for PFNA (CI 5.72–7.51) (Table S5). Increases in PFAS were also associated with higher HDL-C and LDL-C levels, especially for PFOS and PFNA. The sum of PFAS effects (ΣPFAS) was similar to those of PFOA, given its concentration accounted for the majority of the total serum concentration. These trends were confirmed when exposures were considered as categorical (based on quartiles of exposure) (Table S6). Gender significantly modified the association between all four PFASs and HDL-C, whereas the interaction terms for TC and LDL-C were significant only for PFOA and PFHxS (Table S5).

When assessing mixture effects, in the WQS regression models, a one-quartile increase in PFAS mixture was associated with 4.09 mg/dL (95% CI: 3.47–4.71) higher TC after adjusting for all covariates (Table 2). 

A quartile increase in the WQS index was associated with an increase in the HDL-C level of 1.13 mg/dL (95% CI: 0.92–1.33). Finally, for each quartile increase in the WQS index, the concentration of LDL-C increased 3.14 mg/dL (95% CI: 2.65–3.63). We identified PFOS and PFNA as contributing the most to the weighted quantile sum index for TC (Table 2). PFOS showed the highest weight for both HDL-C (0.65) and LDL-C (0.61), indicating its high contribution in driving the observed association with these outcomes, while PFOA and PFHxS had the lowest weights for all outcomes (Table 2). PFOA was identified as a chemical of concern only regarding the association with HDL-C in females (weight: 0.29, Table 2).

Applying Q-Gcomp approach, a one quartile increase in PFAS mixture was associated with 4.04 mg/dL (95% CI: 3.5; 4.58) increase in TC, 1.07 mg/dL increase (95% CI: 0.87; 1.27) in HDL-C and 2.71 mg/dL (95% CI: 2.23; 3.19) in LDL-C (Table 3). 

Both PFOS and PFNA were identified as chemicals of concern when studying the association with TC, while for HDL-C and LDL-C only PFOS exceeded the set threshold. PFOS and PFNA were always weighted positively, among both males and females, suggesting that these compounds are more important drivers of the overall association compared to PFOA and PFHxS (Table 3).

Results from BKMR were displayed as differences in serum lipids levels and 95% confidence intervals when the four PFAS concentrations were at their 75th percentile compared to their medians (Table 4).

The combined effects of the PFAS mixture were associated with increases in all lipid parameters (for TC 2.77 mg/dL with 95% CI: 1.8–3.74; for HDL-C 0.57 mg/dL with 95% CI: 0.42–0.72; and for LDL-C 2.08 mg/dL with 95% CI: 1.15–3.02), and effects were always stronger in females (Table 4). The effects of PFAS mixture on TC, HDL-C and LDL-C displayed linear dose–response relationships overall and in both males and females (Figure S2, Table S7).

PFNA and PFOS had the highest PIPs when studying the association with TC and LDL-C (1 and 1 for both lipid outcomes, respectively), making them the most important group in the mixture, which was in line with the results from WQS and Q-Gcomp approach (Table S8). PFHxS also emerged as an important chemical compound in the association with LDL-C (PIP equal to 1), whereas for HDL-C the PFAS that contributes most in explaining the relationship with the response is PFOS (PIP equal to 1). We further investigated the interactions between a certain PFAS and each lipid parameter when other exposures were fixed at their 50th percentile. No significant changes in the association with TC, HDL-C and LDL-C were found, suggesting no interactions.

## 4. Discussion

The aim of this study was to assess individual and combined effects of exposure to four PFASs on serum lipid profile in more than 34,000 highly exposed adults aged ≥20 years in the Veneto Region, Italy, using different statistical approaches recently developed to specifically quantify the summed mixture effect as well as the contribution of each compound within the mixture. All the selected methods possess strengths and limitations, so it is of interest to compare these approaches in the context of the PFAS-lipids association (Table S2). When the aim is to distinguish the relative importance of components, weighted quantile-based approaches are the most appropriate, as they provide relative weights for each chemical. Not knowing the dose–response function shapes, or the biological mechanism, is a reason for making as few assumptions as possible, leaning towards the use of Q-Gcomp and BKMR. All models (WQS, Q-Gcomp and BKMR analysis) suggested that higher concentrations of mixtures of four PFASs are strongly associated with serum lipids parameters. TC and LDL-C were more strongly associated with exposure to the mixture of PFASs than HDL-C, confirming results from single chemical methods. 

Consensus in the results we obtained through different methods can be attributed to the fact that exposure–outcome curves were generally monotonic and lacked high synergistic effect between individual PFASs. In addition, BKMR analyses supported the associations observed in the linear regression models, suggesting the absence of non-linear associations or interaction effects.

Although the literature on the effect of a single PFAS and lipids is substantial, few studies have examined the combined effects of exposure to PFAS mixtures and lipid outcomes. Similar to the results of the current study, Fan et al. [9] observed a mixture of higher serum PFAS concentrations (PFNA, PFHxS, PFOA and PFOS) associated with higher HDL-C, LDL-C and TC levels in a population of 1067 adults aged ≥20 years with background levels of exposure using both WQS and BKMR analysis. WQS index was significantly correlated with the levels of HDL-C (b: 2.03, 95% CI: 0.74–3.32), LDL-C (b: 4.16, 95% CI: 1.07–7.24) and TC (b: 6.54, 95% CI: 3.00–10.1). In the BKMR analysis, the effect of PFAS on serum lipids increased significantly when the concentrations of the PFAS were at their 60th percentiles or above compared to those at their 50th percentile. In this study, PFHxS had the greatest impact on HDL-C level, and PFNA had the greatest impact on LDL-C and TC levels, the latter being consistent with our findings in the present study. 

A longitudinal study conducted on 306 mother/child pairs from the Shanghai-Minhang Birth Cohort Study [11] drew different conclusions when investigating the effects of prenatal exposure to individual PFASs and PFAS mixtures on cord lipid concentrations using BKMR model. BKMR revealed inverse associations between the mixture of eight PFASs and cord serum lipid concentrations (TC, TG, HDL-C and LDL-C), showing that ln-transformed TC concentrations of exposure to the 75th percentile of the mixture were 0.11 units (95% CI: −0.21; −0.01) lower than the 25th percentile exposure. This generally supported the observed association of individual PFAS exposure (mainly long-chain PFASs (PFNA, PFDA, FFUdA and PFTrDA)) on cord plasma levels of lipid parameters in multiple linear regression models [11]. 

Significantly positive associations were observed between mixtures of PFAS and TC and LDL-C in a case-control study consisting of 304 participants in China, half of which were diagnosed with type 2 diabetes using Q-Gcomp. However, the associations failed to reach significance after adjustment for covariates (age, sex and BMI). In the relations between PFAS and TC, PFUnDA had the greatest positive contribution to the mixture effect on TC, followed by 6:2 Cl-PFESA, PFOA and PFNA, whereas PFDA, PFHxS and PFOS showed negative contributions [26].

In another cross-sectional study on 102 young adults with 82% overweight or obese participants, no association was found for PFAS exposure with serum concentrations of cholesterol and triglycerides using BKMR model [8].

Some aspects of our results deserve to be underlined. First, BKMR, Q-Gcomp and WQS regression identified PFOS and PFNA as the most important contributors in the observed associations of PFAS exposure with all three lipid parameters, though the main exposure in this population was to PFOA (with a median concentration about 23 times higher than the PFOA serum concentration in the Veneto citizens with only background exposures (1.64 ng/mL)) [27]. While PFOS’s strong effect on lipids has been seen in previous studies [7,28], PFNA relevancy in the studied associations was rarely highlighted. The latter already emerged from single-pollutant modelling, emphasizing the greater effect of PFNA despite having remarkably lower changes in concentration than other PFAS.

To the best of our knowledge, we are the first to have assessed the overall association between lipid profile outcomes and PFAS mixture stratified by gender. When stratified by gender in mixture models, the results were in accord with the findings of several other studies using single-pollutant models [7,28,29,30]. Results confirmed a stronger association between overall PFAS mixtures and HDL-C in females, while the association with LDL-C was the strongest in males. PFOA’s major role in describing the association with HDL-C in females emerged consistently, being identified as chemical of concern in both WQS and Q-Gcomp. Compared with men, women at pre-menopause have lower LDL-C levels and higher HDL-C levels [31], indicative of the role that estrogens play in the regulation of the metabolism through receptor modification [32]. This metabolic pattern typical of women could explain the different effect of PFAS on lipids according to sex. Given the apparent impact of PFAS exposure on metabolic health outcomes, future studies are warranted to explore potential sex-specific association between serum-PFAS concentrations and lipid profiles and to explore causal links.

While there is large body of epidemiological studies linking individual PFAS exposure to increased blood lipid levels, mechanistic toxicity studies on individual PFASs, other than PFOA and PFOS, as well as studies that utilize mixtures of PFASs, are limited [33,34]. To date, it is evident that PFOA and to a lesser extent PFOS activate PPARα, both its murine and human version, and the observed lipid alterations may depend to a certain extent on the PPARα-signaling pathway. The stronger association of PFOS and PFNA with total cholesterol and LDL-cholesterol has also been reported in experimental data where serum PFOS and PFNA showed a higher potency based on liver effects than PFOA and PFHxS [35]. 

Among experimental in vitro studies considering a series of PFASs in cultured cells, PFNA was the most potent activator of human and mouse PPAR-α [36], which could explain why its greater contribution than other PFASs emerged using different approaches. PFNA relevancy can be explained by the limited range of variation including a longer persistence in the body and, potentially, a greater intrinsic toxicological power of this PFAS compare to the other PFASs [37]. Recent studies suggest that the positive associations observed in epidemiological studies between PFAS and circulating cholesterol may be more related to impacts on cholesterol metabolism and transport than on endogenous cholesterol formation [34]. 

The present study has several strengths. First, the relatively large sample size, which allowed us to assess these complex relationships while controlling for multiple potential confounding factors and stratifying by sex. To our knowledge, this is the second largest study in a highly exposed population [28]. 

Second, applying statistical methods to assess mixture exposure along with conventional linear regression enabled us to examine both individual and combined effects of the exposure to multiple PFASs, accounting for moderately to highly correlated PFAS concentrations; thus, the potential bias by co-exposed PFASs may be less of a concern in our results. The WQS regression identifies the most important factors with higher accuracy, sensitivity and specificity than single pollutant analyses. However, it is not possible to simultaneously account for any differences in the directions of the associations. Therefore, we introduced quantile G-computation to avoid any a priori constraints on the directionality of the associations. Additionally, the BKMR allowed us to estimate the effect of PFAS mixture without any linearity assumption on the dose–response relationships. Overall, the application of multiple approaches, each with different strengths and limitations, produced consistent results and greatly reduced the chance of false discovery.

Third, through the identification of the chemicals of concern, it was possible to distinguish which PFASs were the most important contributors in the observed associations with serum lipids. However, due to the high correlations between some of the PFAS pairs, the difficulty for distinguishing their independent biological effects remains.

There are some potential limitations to the present study that mainly refer to the study design and are reported in the previous paper [7]. Mainly, our study was cross-sectional, limiting our ability to determine temporality of exposures in relation to the outcomes, but this is potentially offset by the relatively long half-lives of the investigated PFASs in blood. While we were able to assess potential confounding by numerous socio-demographic factors, we cannot rule out potential residual confounding from uncontrolled factors. In addition, the associations of exposure to PFASs with serum lipid profiles may be affected by other chemicals which correlated with PFAS exposure, thus we could not exclude the potential effects of other chemicals co-occurring with PFAS exposure in our study.

## 5. Conclusions

In this large study, we found that PFAS exposure, especially PFOS and PFNA exposures, has a significant association with serum lipid parameters in the highly exposed adult population of the Veneto Region, Italy. The mixture of the four PFASs with higher concentrations in blood samples was positively associated with TC, HDL-C and LDL-C, whether applying WQS, Q-Gcomp or BKMR modelling. These results suggest that the evaluation of multichemical exposure could better characterize their real toxicity compared with single-chemical exposure assessment, due to the fact that PFASs occur as mixtures in the environment. Given the ubiquitous nature of PFASs, more effort is needed in future studies to understand the potential effects of their exposure in potentially vulnerable population subgroups.

## Figures and Tables

**Table 1:** Distributions of serum PFASs concentrations (ng/mL) and serum lipids concentrations (mg/dL) in the study population (*n* = 34,633), stratified by gender.

	Males					Females					Total				
	Mean (SD)	GM	Min–Max	Median (Q1–Q3)	<LOQ	Mean (SD)	GM	Min–Max	Median (Q1–Q3)	<LOQ	Mean (SD)	GM	Min–Max	Median (Q1–Q3)	<LOQ
PFAS															
PFOA	90.5 (100.4)	52.7	0.4–2723.3	60.7 (26.0–120.7)	0.05%	39.2 (47.05)	21.5	0.4–1090.0	24.1 (9.7–51.5)	0.22%	63.7 (81.1)	32.8	0.4–2723.3	37.0 (14.6–82.3)	0.14%
PFOS	6.2 (4.7)	5.1	0.4–142.0	5.10 (3.4–7.5)	0.13%	4.0 (3.49)	3.2	0.4–124.0	3.2 (2.2–4.9)	0.37%	5.0 (4.2)	4.0	0.4–142.0	4.0 (2.6–6.2)	0.26%
PFHxS	10.2 (9.8)	6.6	0.4–162.0	7.40 (3.4–13.8)	0.64%	3.7 (3.88)	2.4	0.4–71.0	2.5 (1.2–4.8)	5.31%	6.7 (7.9)	3.9	0.4–162.0	4.0 (1.8–8.8)	3.11%
PFNA	0.6 (0.6)	0.6	0.4–59.8	0.50 (0.4–0.7)	33.03%	0.5 (0.32)	0.5	0.4–12.3	0.4 (0.4–0.6)	59.57%	0.6 (0.5)	0.5	0.4–59.8	0.5 (0.4–0.7)	47.07%
Outcome															
TC	191.0 (38.3)		70–487	188 (164–215)		188.3 (36.2)		83–450	185 (163–211)		189.6 (37.2)		70–487	187 (163–213)	
HDL-C	52.8 (12.4)		15–138	52 (44–60)		64.6 (14.5)		22–144	63 (55–73)		59.1 (14.7)		15–144	58 (48–68)	
LDL-C	114.4 (34.5)		0–420	112 (91–136)		106.2 (31.5)		0–342	103 (84–125)		110.0 (33.2)		0–420	107 (87–131)	

Note: total cholesterol (TC), high-density lipoprotein cholesterol (HDL-C) and low-density lipoprotein cholesterol (LDL-C).

**Table 2:** Associations between WQS regression index and serum lipids (mg/dL). WQS estimates * and 95% confidence intervals for the change in TC, HDL-C and LDL-C, stratified by gender. WQS regression model weights of each PFAS component, for each outcome.

	Outcome	β [95% CI]	Weights			
			PFOA	PFOS	PFHxS	PFNA
Males	TC	**3.63** [2.88; 4.39]	0	**0.67**	0	**0.33**
	HDL-C	**0.7** [0.46; 0.95]	0	**0.75**	0	**0.25**
	LDL-C	**3.46** [2.70; 4.22]	0	**0.39**	0	**0.61**
Females	TC	**4.61** [3.54; 5.67]	0.18	**0.31**	0	**0.52**
	HDL-C	**1.61** [1.25; 1.97]	**0.29**	**0.37**	0.03	**0.31**
	LDL-C	**2.57** [1.89; 3.24]	0.03	**0.63**	0.01	**0.33**
Total	TC	**4.09** [3.47; 4.71]	0.14	**0.43**	0	**0.43**
	HDL-C	**1.13** [0.92; 1.33]	0.12	**0.65**	0	**0.23**
	LDL-C	**3.14** [2.65; 3.63]	0.02	**0.61**	0	**0.37**

Note: β represents the increase of cholesterol level associated with a quartile increase in the WQS index. In bold: weights that exceed the case of uniform weights (>1/*p* = 0.25). * adjusted by age, BMI, time-lag between the enrolment and the beginning of the study and categorical covariates including sex, smoking habits, country of birth, alcohol consumption, education level, laboratory in charge of the analyses of serum lipids and reported food consumption (in tertiles or quartiles of fruit/vegetables, milk/yogurt, cheese, meat, sweet/snacks/sweet beverage, eggs, fish, bread/pasta/cereals per week).

**Table 3:** Associations between Quantile G-computation regression index and serum lipids (mg/dL). Quantile G-computation estimates * and 95% confidence intervals for the change in TC, HDL-C and LDL-C, stratified by sex. Q-Gcomp regression model weights of each PFAS component, for each outcome.

	Outcome	ψ [95% CI]	Weights					
			Direction	Effect	PFOA	PFOS	PFHxS	PFNA
Males	TC	**3.14** [2.39; 3.9]	pos	4.56	0.01	**0.59**		**0.40**
			neg	−1.42			1.00	
	HDL-C	**0.56** [0.30; 0.81]	pos	0.84		**0.60**		0.40
			neg	−0.28	0.26		0.74	
	LDL-C	**2.35** [1.67; 3.03]	pos	4.00		**0.63**		0.37
			neg	−1.65	0.49		0.51	
Females	TC	**4.82** [3.56; 6.08]	pos	5.29	0.28	**0.35**		**0.37**
			neg	−0.46			1.00	
	HDL-C	**1.58** [1.05; 2.12]	pos	1.94	**0.35**	**0.39**		0.26
			neg	−0.35			1.00	
	LDL-C	**2.59** [1.51; 3.68]	pos	2.83	0.12	**0.57**		0.31
			neg	−0.23			1.00	
Total	TC	**4.04** [3.50; 4.58]	pos	4.74	0.16	**0.44**		**0.40**
			neg	−0.70			1.00	
	HDL-C	**1.07** [0.87; 1.27]	pos	1.29	0.28	**0.45**		0.26
			neg	−0.22			1.00	
	LDL-C	**2.71** [2.23; 3.19]	pos	3.29		**0.57**		0.43
			neg	−0.58	0.48		0.52	

Note: ψ represents the increase of cholesterol level associated with a quartile increase within the overall mixture, according to quantile G-computation. In bold: weights that exceed the case of uniform positive weights (>1/*p*, where p represents the number of chemicals with positive weights in each group). * adjusted by age, BMI, time-lag between the enrolment and the beginning of the study, and categorical covariates including sex, smoking habits, country of birth, alcohol consumption, education level, laboratory in charge of the analyses of serum lipids and reported food consumption (in tertiles or quartiles of fruit/vegetables, milk/yogurt, cheese, meat, sweet/snacks/sweet beverage, eggs, fish, bread/pasta/cereals per week).

**Table 4:** BKMR estimates * and 95% confidence intervals for the change in TC, HDL-C and LDL-C, stratified by sex.

	Outcome	δ^50–75^ (95% CI)
Males	TC	2.34 [1.61; 3.07]
	HDL-C	0.49 [0.33; 0.66]
	LDL-C	1.35 [0.75; 1.95]
Females	TC	4.25 [3.30; 5.20]
	HDL-C	1.36 [0.88; 1.84]
	LDL-C	1.82 [0.85; 2.79]
Total	TC	2.77 [1.80; 3.74]
	HDL-C	0.57 [0.42; 0.72]
	LDL-C	2.08 [1.15; 3.02]

Note: δ^50–75^ represents the increase of cholesterol level associated with a quartile increase within the overall effect when PFAS mixtures were at their 75th percentiles compared to their median, according to BKMR. * adjusted by age, BMI, time-lag between the enrolment and the beginning of the study, and categorical covariates including sex, smoking habits, country of birth, alcohol consumption, education level, laboratory in charge of the analyses of serum lipids and reported food consumption (in tertiles or quartiles of fruit/vegetables, milk/yogurt, cheese, meat, sweet/snacks/sweet beverage, eggs, fish, bread/pasta/cereals per week).

## Data Availability

The data are not publicly available. The data presented in this study are available on request from the corresponding author.

## Supplementary Materials

The following supporting information can be downloaded at: https://www.mdpi.com/article/10.3390/ijerph191912421/s1, Figure S1: Flow-chart of study population; Table S1: Distributions of serum PFASs concentrations (ng/mL) excluded from the main analyses in the study population (n = 34,633); Table S2: Principal characteristics of recently developed statistical methods used for mixture analysis; Table S3: Distributions of covariates in the study population (n = 34,633); Table S4: Spearman correlation matrix of serum PFAS in the study population (n = 34,633), stratified by gender; Table S5: Association between PFAS (ln ng/mL) and Serum Lipids (mg/dL) from GAM models, stratified by gender and adjusted by several covariates: β coefficients for ln-transformed PFAS and 95% Confidence Intervals (CI); Table S6: Association between PFAS (ln ng/mL) and Serum Lipids (mg/dL) from GAM models adjusted by several covariates, β coefficients for PFAS quartiles and 95% Confidence Intervals (CI); Figure S2: The overall effect of the PFAS mixture on serum lipid profiles (95% CIs), estimated using BKMR modeling; Table S7: The overall effect of the PFAS mixture on serum lipid profiles (95% CIs), estimated using BKMR modeling; Table S8: Posterior inclusion probability of each PFAS, stratified by gender.
