# Supplementary material for: Associations between Mixture of Perfluoroalkyl Substances and Lipid Profile in a Highly Exposed Adult Community in the Veneto Region
Source: Int J Environ Res Public Health. 2022 Sep 29;19(19):12421. doi: 10.3390/ijerph191912421 (PMC9566306; doi:10.3390/ijerph191912421)
Supplement: Supplementary file 1 [file ijerph-19-12421-s001.zip › ijerph-1904529-supplementary.pdf]

SUPPLMENTARY MATERIALS

Figure S1. Flow-chart of study population.

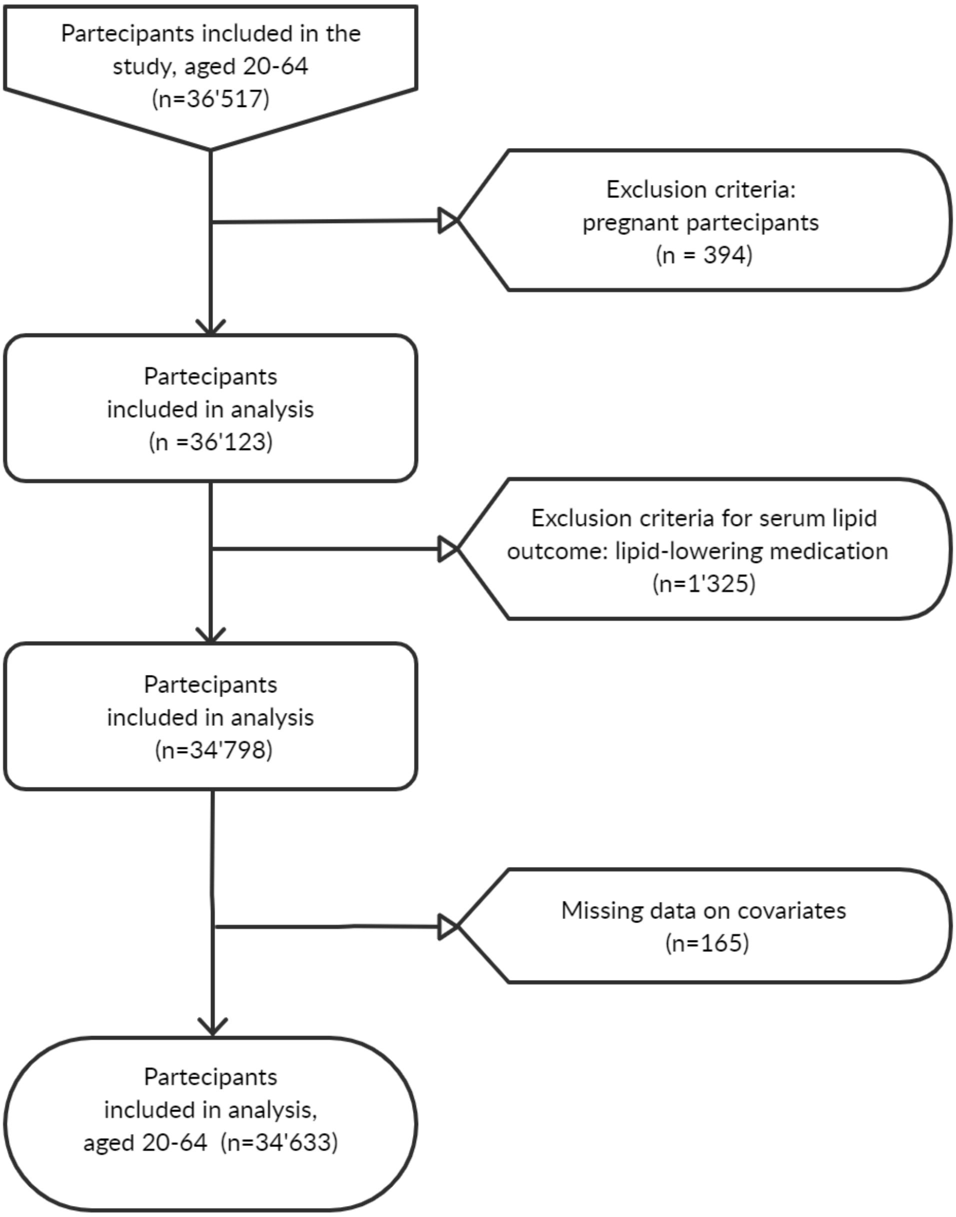

Table S1. Distributions of serum PFASs concentrations (ng/mL) excluded from the main analyses in the study population (n=34,633).

PFAS	Mean (SD)	GM	Min-Max	Median (Q1-Q3)	<LOQ
PFHpA	0.36 (0.15)	0.36	0.35-15.10	0.35 (0.35-0.35)	98.50%
PFBS	0.36 (0.09)	0.36	0.35-5.80	0.35 (0.35-0.35)	78.00%
PFHxA	0.35 (0.04)	0.35	0.35-7.10	0.35 (0.35-0.35)	99.60%
PFBA	0.36 (0.16)	0.36	0.35-23.90	0.35 (0.35-0.35)	99.30%
PFPeA	0.35 (0.00)	0.35	0.35-0.60	0.35 (0.35-0.35)	99.90%
PFDeA	0.44 (0.41)	0.40	0.35-45.30	0.35 (0.35-0.35)	99.90%
PFUnA	0.37 (0.08)	0.36	0.35-6.00	0.35 (0.35-0.35)	95.40%
PFDoA	0.36 (0.08)	0.35	0.35-10.60	0.35 (0.35-0.35)	99.10%

Table S2. Principal characteristics of recently developed statistical methods used for mixture analysis.

Method	WQS	Q-gcomp	BKMR
Overall effect	yes	yes	yes
Individual effect	no	no	yes
Contribution to the mixture	yes	yes	no
Allows non-linearity	no	yes	yes
Allows interactions	no	yes	yes
Selection of the directionality not required	no	yes	yes
R package	gWQS	qgcomp	bkmr
Reference	(Carrico et al., 2015)	(Keil et al., 2020)	(Bobb et al., 2015)

Table S3. Distributions of covariates in the study population (n=34,633).

**Characteristics**				
		**Mean (SD)**	**Min-Max**	**Median (Q1-Q3)**
**Age (years)**		39.95 (10.98)	20 - 64	41 (31 - 49)
**BMI**		24.86 (4.61)	13.39 - 61.72	24.17 (21.62 - 27.18)
**Time-lag between the beginning of the study and blood sampling (months)**		22.32 (9.56)	3 - 42	21 (14 - 32)
		n	***%***	
**Gender**	Females	18,320	52.90%	
	Males	16,313	47.10%	
**Country of birth**	HDC	31,529	91.04%	
	HMPC	3,104	8.96%	
**Smoking habit**	Non-smoker	20,700	59.77%	
	Current smoker	7,806	22.54%	
	Previous smoker	6,127	17.69%	
**Alcohol intake (AU per week)**	None	9,785	28.25%	
	1-2	12,143	35.06%	
	3-6	6,062	17.50%	
	>7	6,643	19.18%	
**Education**	Elementary/Middle school	10,481	30.26%	
	Highschool	17,741	51.23%	
	University	6,411	18.51%	
**Laboratory**	Arzignano	19,412	56.05%	
	Legnago	8,047	23.24%	
	San Bonifacio	7,174	20.71%	

Table S4. Spearman correlation matrix of serum PFAS in the study population (n=34,633), stratified by gender.

**Males**					**Females**					**Total**				
**PFAS**	*PFOA*	*PFOS*	*PFHXS*	*PFNA*	**PFAS**	*PFOA*	*PFOS*	*PFHXS*	*PFNA*	**PFAS**	*PFOA*	*PFOS*	*PFHXS*	*PFNA*
PFOA	1.00				*PFOA*	1.00				*PFOA*	1.00			
PFOS	0.50	1.00			*PFOS*	0.59	1.00			*PFOS*	0.60	1.00		
PFHxS	0.87	0.54	1.00		*PFHxS*	0.90	0.63	1.00		*PFHxS*	0.90	0.65	1.00	
PFNA	0.33	0.68	0.32	1.00	*PFNA*	0.37	0.61	0.37	1.00	*PFNA*	0.41	0.68	0.42	1.00

Table S5. Association between PFAS (ln ng/mL) and Serum Lipids (mg/dL) from GAM models, stratified by gender and adjusted by several covariates: β coefficients for ln-transformed PFAS and 95% Confidence Intervals (CI).*

TC							
**PFAS**	**Males**		**Females**		**Total**		***p-value***
	**β**	**CI**	**β**	**CI**	**β**	**CI**	***log_PFAS:sexM***
PFOA	**1.26**	[0.77 ; 1.75]	**1.76**	[1.34 ; 2.18]	**1.83**	[1.51 ; 2.15]	**<0.001**
PFOS	**5.23**	[4.35 ; 6.12]	**4.13**	[3.37 ; 4.88]	**5.14**	[4.56 ; 5.72]	0.188
PFHXS	**0.75**	[0.18 ; 1.32]	**1.86**	[1.35 ; 2.38]	**1.74**	[1.36 ; 2.13]	**<0.001**
PFNA	**6.99**	[5.70 ; 8.28]	**4.78**	[3.54 ; 6.02]	**6.61**	[5.72 ; 7.51]	0.801
∑PFAS	**1.61**	[1.05 ; 2.17]	**2.24**	[1.74 ; 2.74]	**2.3**	[1.93 ; 2.68]	**<0.001**
**HDL-C**							
**PFAS**	**Males**		**Females**		**Total**		***p-value***
	**β**	**CI**	**β**	**CI**	**β**	**CI**	***log_PFAS:sexM***
PFOA	-0.01	[-0.17 ; 0.15]	**0.60**	[0.42 ; 0.78]	**0.32**	[0.20 ; 0.44]	**<0.001**
PFOS	**0.97**	[0.68 ; 1.27]	**1.66**	[1.34 ; 1.98]	**1.34**	[1.12 ; 1.56]	**<0.001**
PFHXS	-0.04	[-0.23 ; 0.15]	**0.65**	[0.43 ; 0.86]	**0.31**	[0.16 ; 0.45]	**<0.001**
PFNA	**1.21**	[0.78 ; 1.65]	**1.67**	[1.15 ; 2.19]	**1.43**	[1.09 ; 1.76]	**0.017**
∑PFAS	0.03	[-0.16 ; 0.22]	**0.84**	[0.63 ; 1.05]	**0.45**	[0.31 ; 0.59]	**<0.001**
**LDL-C**							
**PFAS**	**Males**		**Females**		**Total**		***p-value***
	**β**	**CI**	**β**	**CI**	**β**	**CI**	***log_PFAS:sexM***
PFOA	**0.75**	[0.31 ; 1.18]	**0.92**	[0.55 ; 1.28]	**1.10**	[0.81 ; 1.38]	**<0.001**
PFOS	**4.58**	[3.79 ; 5.38]	**2.88**	[2.23 ; 3.54]	**4.11**	[3.60 ; 4.62]	0.2
PFHXS	**0.57**	[0.05 ; 1.08]	**1.07**	[0.63 ; 1.51]	**1.22**	[0.88 ; 1.55]	**<0.001**
PFNA	**5.75**	[4.59 ; 6.92]	**3.17**	[2.10 ; 4.24]	**5.12**	[4.33 ; 5.91]	0.126
∑PFAS	**1.04**	[0.54 ; 1.55]	**1.19**	[0.76 ; 1.62]	**1.45**	[1.12 ; 1.78]	**<0.001**

**adjusted by age, BMI, time-lag between the enrolment and the beginning of the study and categorical covariates including sex, smoking habits, country of birth, alcohol consumption, education level, laboratory in charge of the analyses of serum lipids and reported food consumption (in tertiles or quartiles of fruit/ vegetables, milk/yogurt, cheese, meat, sweet/snacks/sweet beverage, eggs, fish, bread/pasta/cereals per week.*

Table S6. Association between PFAS (ln ng/mL) and Serum Lipids (mg/dL) from GAM models adjusted by several covariates, β coefficients for PFAS quartiles and 95% Confidence Intervals (CI).*

PFAS	Quartile	Total C		HDL-C		LDL-C	
		β	CI 95%	β	CI 95%	β	CI 95%
PFOA	Q1 [0.354,14.6]	194.87		62.01		114.54	
	Q2 (14.6,37]	**2.17**	[1.18 ; 3.16]	**0.49**	[0.12 ; 0.86]	**1.16**	[0.28 ; 2.03]
	Q3 (37,82.3]	**4.31**	[3.27 ; 5.34]	**1.12**	[0.73 ; 1.51]	**2.59**	[1.68 ; 3.50]
	Q4 (82.3,2720]	**5.51**	[4.39 ; 6.63]	**1.06**	[0.64 ; 1.48]	**3.29**	[2.30 ; 4.27]
	per IQR**	**1.87**	[1.51 ; 2.23]	**0.38**	[0.25 ; 0.52]	**1.13**	[0.82 ; 1.45]
PFOS	Q1 [0.354,2.6]	194.47		61.78		113.84	
	Q2 (2.6,4]	**3.38**	[2.40 ; 4.37]	**1.06**	[0.69 ; 1.43]	**2.56**	[1.69 ; 3.43]
	Q3 (4,6.2]	**5.68**	[4.67 ; 6.70]	**1.58**	[1.20 ; 1.96]	**4.24**	[3.34 ; 5.13]
	Q4 (6.2,142]	**8.21**	[7.13 ; 9.29]	**2.17**	[1.76 ; 2.58]	**6.58**	[5.63 ; 7.54]
	per IQR**	**2.7**	[2.35 ; 3.04]	**0.7**	[0.57 ; 0.83]	**2.14**	[1.84 ; 2.45]
PFHxS	Q1 [0.354,1.8]	194.85		62.03		114.32	
	Q2 (1.8,4]	**2.89**	[1.90 ; 3.88]	**0.64**	[0.26 ; 1.01]	**1.99**	[1.12 ; 2.86]
	Q3 (4,8.8]	**4.25**	[3.22 ; 5.29]	**1.05**	[0.66 ; 1.44]	**2.68**	[1.77 ; 3.59]
	Q4 (8.8,162]	**4.57**	[3.41 ; 5.73]	**0.88**	[0.44 ; 1.31]	**3.22**	[2.20 ; 4.24]
	per IQR**	**1.54**	[1.17 ; 1.91]	**0.32**	[0.18 ; 0.46]	**0.54**	[0.42 ; 0.66]
PFNA	Q1 [0.354]	195.07		62.10		114.33	
	Q2 (0.354,0.5]	**3.51**	[2.49 ; 4.54]	**0.71**	[0.32 ; 1.09]	**2.59**	[1.69 ; 3.49]
	Q3 (0.5,0.7]	**5.20**	[4.25 ; 6.16]	**1.13**	[0.77 ; 1.49]	**3.88**	[3.03 ; 4.72]
	Q4 (0.7,59.8]	**7.32**	[6.29 ; 8.35]	**1.61**	[1.23 ; 2.00]	**5.77**	[4.86 ; 6.67]
	per IQR**	**2.47**	[2.15 ; 2.78]	**1.06**	[0.73 ; 1.38]	**1.92**	[1.64 ; 2.2]
∑PFAS	Q1 [4.25,20.8]	194.72		61.76		113.27	
	Q2 (20.8,46.9]	**2.83**	[1.83 ; 3.82]	**0.80**	[0.43 ; 1.17]	**1.75**	[0.87 ; 2.63]
	Q3 (46.9,98]	**4.82**	[3.78 ; 5.87]	**1.21**	[0.82 ; 1.60]	**3.08**	[2.16 ; 4.00]
	Q4 (98,2930]	**6.02**	[4.89 ; 7.15]	**1.26**	[0.84 ; 1.68]	**3.72**	[2.73 ; 4.71]
	per IQR**	**2.01**	[1.65 ; 2.37]	**0.42**	[0.28 ; 0.55]	**1.25**	[0.93 ; 1.57]

Note: The first β coefficient for each PFAS is the predicted values of each outcome for the 1st percentile (quartile) of the PFAS distribution.

**adjusted by age, BMI, time-lag between the enrolment and the beginning of the study and categorical covariates including sex, smoking habits, country of birth, alcohol consumption, education level, laboratory in charge of the analyses of serum lipids and reported food consumption (in tertiles or quartiles of fruit/ vegetables, milk/yogurt, cheese, meat, sweet/snacks/sweet beverage, eggs, fish, bread/pasta/cereals per week.*

*** coefficient for an interquartile range (IQR) increment*

Figure S2. The overall effect of the PFAS mixture on serum lipid profiles (95% CIs), estimated using BKMR modeling.*

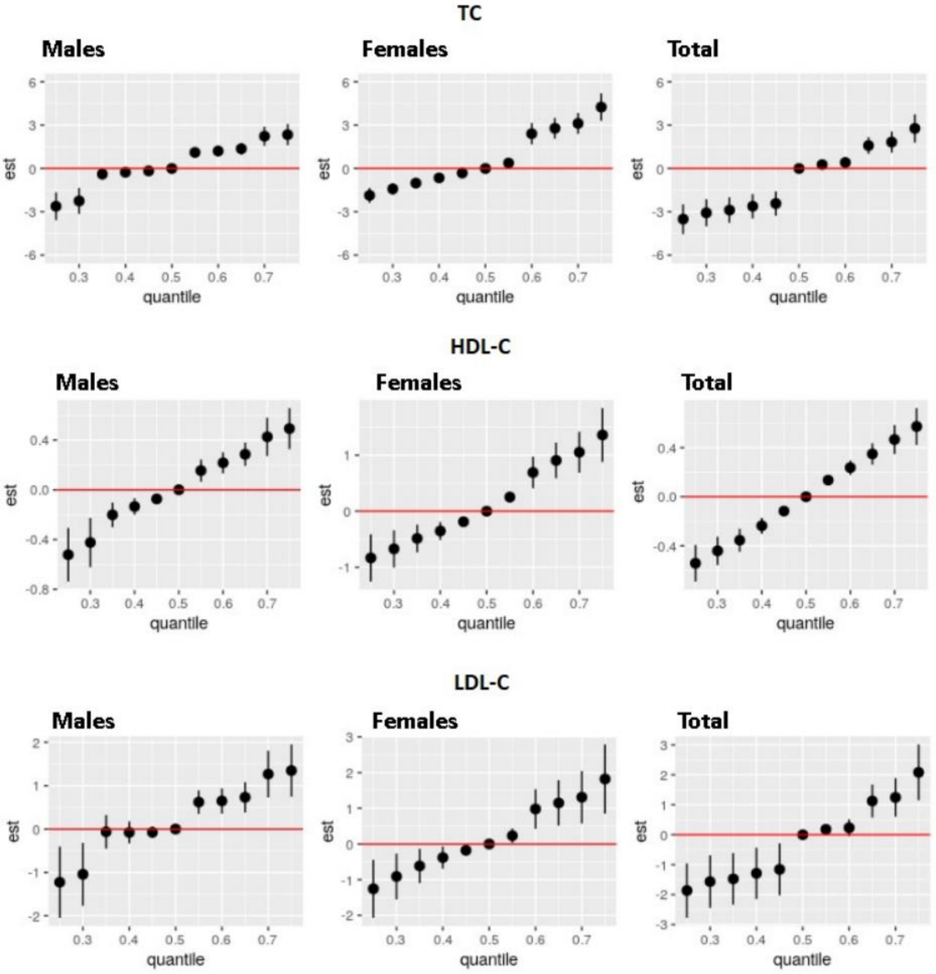

Note: This figure showed the overall effect of the PFAS mixture on serum lipid profiles (estimates and 95% confidence intervals) when PFAS mixtures were at a particular percentile (x-axis) compared to when PFAS mixtures were all at their 50th percentiles by BKMR model. The overall effect of the PFAS mixtures were shown for TC, HDL-C and LDL-C.

**adjusted by age, BMI, time-lag between the enrolment and the beginning of the study and categorical covariates including sex, smoking habits, country of birth, alcohol consumption, education level, laboratory in charge of the analyses of serum lipids and reported food consumption (in tertiles or quartiles of fruit/ vegetables, milk/yogurt, cheese, meat, sweet/snacks/sweet beverage, eggs, fish, bread/pasta/cereals per week.*

Table S7. The overall effect of the PFAS mixture on serum lipid profiles (95% CIs), estimated using BKMR modeling.*

TC						
quantile	Males		Females		Total	
	estimate	s.e.	estimate	s.e.	estimate	s.e.
0.25	**-2.62**	0.49	**-1.86**	0.26	**-3.51**	0.53
0.30	**-2.25**	0.46	**-1.41**	0.20	**-3.07**	0.48
0.35	**-0.39**	0.20	**-1.01**	0.15	**-2.88**	0.45
0.40	**-0.26**	0.13	**-0.65**	0.10	**-2.62**	0.43
0.45	**-0.17**	0.07	**-0.32**	0.05	**-2.43**	0.43
0.50	0.00	0.00	0.00	0.00	0.00	0.00
0.55	**1.11**	0.18	**0.38**	0.06	**0.27**	0.11
0.60	**1.21**	0.18	**2.41**	0.38	**0.41**	0.19
0.65	**1.37**	0.21	**2.78**	0.37	**1.59**	0.29
0.70	**2.23**	0.34	**3.12**	0.37	**1.83**	0.38
0.75	**2.34**	0.37	**4.25**	0.48	**2.77**	0.50

HDL						
quantile	Males		Females		Total	
	estimate	s.e.	estimate	s.e.	estimate	s.e.
0.25	**-0.52**	0.11	**-0.83**	0.21	**-0.54**	0.08
0.30	**-0.42**	0.10	**-0.67**	0.17	**-0.44**	0.06
0.35	**-0.20**	0.05	**-0.49**	0.13	**-0.35**	0.05
0.40	**-0.13**	0.03	**-0.35**	0.08	**-0.24**	0.03
0.45	**-0.07**	0.02	**-0.19**	0.04	**-0.12**	0.02
0.50	0.00	0.00	0.00	0.00	0.00	0.00
0.55	**0.15**	0.05	**0.25**	0.05	**0.14**	0.02
0.60	**0.22**	0.04	**0.69**	0.14	**0.24**	0.03
0.65	**0.29**	0.05	**0.91**	0.16	**0.35**	0.04
0.70	**0.43**	0.08	**1.05**	0.19	**0.47**	0.06
0.75	**0.49**	0.08	**1.36**	0.24	**0.57**	0.08

LDL						
quantile	Males		Females		Total	
	estimate	s.e.	estimate	s.e.	estimate	s.e.
0.25	**-1.23**	0.42	**-1.26**	0.41	**-1.87**	0.46
0.30	**-1.04**	0.37	**-0.91**	0.33	**-1.57**	0.45
0.35	-0.06	0.20	**-0.62**	0.25	**-1.47**	0.44
0.40	-0.08	0.13	**-0.38**	0.16	**-1.29**	0.44
0.45	-0.08	0.07	**-0.18**	0.08	**-1.16**	0.44
0.50	0.00	0.00	0.00	0.00	0.00	0.00
0.55	**0.62**	0.14	**0.23**	0.10	**0.18**	0.08
0.60	**0.65**	0.15	**0.98**	0.28	0.23	0.14
0.65	**0.73**	0.18	**1.15**	0.32	**1.13**	0.28
0.70	**1.27**	0.27	**1.31**	0.38	**1.25**	0.33
0.75	**1.35**	0.31	**1.82**	0.49	**2.08**	0.48

Note: The overall effect of the PFAS mixture on serum lipid profiles (estimates and standard errors) when PFAS mixtures were at a particular percentile (x-axis) compared to when PFAS mixtures were all at their 50th percentiles are shown.
**adjusted by age, BMI, time-lag between the enrolment and the beginning of the study and categorical covariates including sex, smoking habits, country of birth, alcohol consumption, education level, laboratory in charge of the analyses of serum lipids and reported food consumption (in tertiles or quartiles of fruit/ vegetables, milk/yogurt, cheese, meat, sweet/snacks/sweet beverage, eggs, fish, bread/pasta/cereals per week.*

Table S8. Posterior inclusion probability of each PFAS, stratified by gender.

TC					
Males		Females		Total	
variable	PIP	variable	PIP	variable	PIP
PFOA	0.78	PFOA	0.3	PFOA	0
PFOS	1	PFOS	1	PFOS	1
PFHxS	1	PFHxS	0	PFHxS	0.84
PFNA	1	PFNA	1	PFNA	1
HDL-C					
Males		Females		Total	
variable	PIP	variable	PIP	variable	PIP
PFOA	0	PFOA	1	PFOA	0.58
PFOS	1	PFOS	1	PFOS	1
PFHxS	0	PFHxS	1	PFHxS	0.68
PFNA	0.2	PFNA	1	PFNA	0.68
LDL-C					
Males		Females		Total	
variable	PIP	variable	PIP	variable	PIP
PFOA	0.14	PFOA	0.1	PFOA	0.94
PFOS	1	PFOS	1	PFOS	1
PFHxS	1	PFHxS	0.8	PFHxS	1
PFNA	1	PFNA	0.74	PFNA	1
